# Microscale Testing and Modelling of Cement Paste as Basis for Multi-Scale Modelling

**DOI:** 10.3390/ma9110907

**Published:** 2016-11-08

**Authors:** Hongzhi Zhang, Branko Šavija, Stefan Chaves Figueiredo, Mladena Lukovic, Erik Schlangen

**Affiliations:** Faculty of Civil Engineering and Geosciences, Delft 2628 CN, The Netherlands; b.savija@tudelft.nl (B.Š.); s.chavesfigueiredo@tudelft.nl (S.C.F.); m.lukovic@tudelft.nl (M.L.); erik.schlangen@tudelft.nl (E.S.)

**Keywords:** micro-mechanics, fracture, X-ray computed tomography, lattice model

## Abstract

This work aims to provide a method for numerically and experimentally investigating the fracture mechanism of cement paste at the microscale. For this purpose, a new procedure was proposed to prepare micro cement paste cubes (100 × 100 × 100 µm^3^) and beams with a square cross section of 400 × 400 µm^2^. By loading the cubes to failure with a Berkovich indenter, the global mechanical properties of cement paste were obtained with the aid of a nano-indenter. Simultaneously the 3D images of cement paste with a resolution of 2 µm^3^/voxel were generated by applying X-ray microcomputed tomography to a micro beam. After image segmentation, a cubic volume with the same size as the experimental tested specimen was extracted from the segmented images and used as input in the lattice model to simulate the fracture process of this heterogeneous microstructure under indenter loading. The input parameters for lattice elements are local mechanical properties of different phases. These properties were calibrated from experimental measured load displacement diagrams and failure modes in which the same boundary condition as in simulation were applied. Finally, the modified lattice model was applied to predict the global performance of this microcube under uniaxial tension. The simulated Young’s modulus agrees well with the experimental data. With the method presented in this paper the framework for fitting and validation of the modelling at microscale was created, which forms a basis for multi-scale analysis of concrete.

## 1. Introduction

Cement based materials are the dominant construction materials in the world [1]. Since cement paste is the most basic and complex component of these materials, the understanding of its mechanical properties and fracture behaviour is of significant practical importance and scientific interest. Contrary to homogenous isotropic materials, the stress field inside this highly heterogeneous material is not uniform even under uniform loading, which leads to microcracking at a number of locations prior to crack localization [2]. These microcracks eventually develop and coalesce to form a critical macrocrack leading to the failure of this material at a low strain level.

Since the critical scale for studying and understanding the fracture behaviour of cement paste is the microscale [2], researchers have tried various methods to simulate the fracture performance and mechanical properties of cement at this scale. In these approaches, two aspects should be included. The first one is how to obtain a realistic microstructure. This can be achieved by numerical models or experiments. Compared with experiments, the computer generated microstructural models, such as cement hydration model, are easier and faster. However, cement particles are commonly simulated as spheres [3,4,5], which will influence the simulated hydration of cement [6]. Therefore, although the experimentally obtained microstructure has a disadvantage in terms of spatial resolution, the realistic particle shape and phase distribution after the onset of hydration can be captured. Over the past decades, huge advances have been made in microstructural characterisation techniques. A good resolution of the microstructure can be provided by using backscattered electron imaging (BSE) in the scanning electron microscope (SEM) [7]. However, the main shortcoming of this technique is the lack of 3D information. To overcome this, X-ray microcomputed tomography (μCT), which provides a non-destructive way of obtaining three dimensional information on the interior of materials, has been applied in the study of cement based materials over the last two decades [8,9,10]. By applying synchrotron radiation as the X-ray source, an improved resolution of between 0.5 and 1.0 μm for cement-based materials can be achieved [11,12,13], which provides very detailed information about the 3D microstructural evolution of these materials.

The second aspect is how to include the local micromechanical properties of different phases in cement paste. Usually micromechanical properties of different phases are derived from standard nano-indentation measurements [14,15,16,17]. Meanwhile, an alternative way is to use molecular dynamics simulations to calculate the micromechanical properties of cement phases, e.g., Calcium-Silicate-Hydrate (C–S–H) [18,19]. Once the microstructure and the micromechanical properties are available, the micromechanical modelling approaches can be applied to simulate the fracture behaviour and mechanical properties of cement paste. Generally, the micromechanical modelling approaches can be classified in two categories: continuum approaches and discrete (lattice) approaches. Although continuum approaches are more widely used to predict the elastic properties of cement paste [20,21,22,23], they have inherent difficulties when dealing with strain localization (fracture) processes. On the other hand, lattice models show a great advantage because not only the stress-strain response, but also cracks pattern and microcracks propagation, can be simulated [24,25,26]. Continuum material behaviour can be, with certain limitations, reproduced by this class of models [27,28]. In lattice models, the continuum is replaced by a lattice system of beam elements and the crack growth is realized by using a sequentially-linear solution procedure [29]. This procedure implies performing a linear elastic analysis in every step; then, a single element with the highest stress/strength ratio is identified and removed from the mesh, thereby introducing a discontinuity; this procedure is then repeated until a global failure criterion is reached. Recently, the fracture process of micro cement paste cubes under uniaxial tension was simulated by Qian et al. [30] and Lukovic et al. [31] using the 3D lattice model. In spite of the similar applied simulation strategies, the obtained mechanical properties of cement paste are still not reliable because the relationship between the indentation hardness and the tensile strength (which is one of the main inputs for the model) of individual phases in hydrated cement paste is not known at present. Since inverse calculation of these local properties is possible only when an experiment on specimen size is available and uniaxial tensile testing on the micro-length scale is still impossible to achieve [2], an important question arising here is how to validate the modelling results.

Recently, a new method named microcube indentation has been developed to test the global mechanical performance of micro cement cubes (100 × 100 × 100 µm^3^) using nano-indenter, which provides an unprecedented opportunity for validation of mechanical simulation results at the microscale [32]. The new method uses nano-indentation equipment to assess the fracture properties of small specimens, unlike regular nano-indentation testing that is used to assess elastic modulus and hardness. This method is further developed and presented in this paper. The method for experimental testing and numerical simulation of specimens on the same size under the same boundary conditions is addressed. The adopted mechanical properties of local phases are evaluated by comparing the simulated damage evolution and load displacement diagram with the experimental observation. In addition, the calibrated model is applied to predict the global mechanical properties of micro cement cube under uniaxial tension. The predicted results are compared with the results of the previous works.

## 2. Experimental

### 2.1. Sample Preparation

The following section describes the procedure to prepare microcubes (100 × 100 × 100 µm^3^) for global mechanical performance test and microbeams with a cross section of 400 × 400 µm^2^ for CT scan. Standard grade OPC CEM I 42.5 N cement paste with w/c ratio 0.40 was cast in a PVC cylinder (diameter, 24 mm, height 39 mm) in sealed condition. After 24 h rotation and curing 28 days at room temperature (20 °C), specimens were demoulded and two discs with the thickness of 2 mm were cut from the middle part. One of the pieces was used to create the microcubes, while the other was used to create the microbeams. The hydration was arrested by solvent exchange method using isopropanol [33]. In order to enable faster water-solvent exchange, samples were immerged five times and taken out for a period of one minute. Afterwards, they were placed for 3 days in isopropanol and subsequently taken out, and solvent was removed by evaporation at ambient conditions.

**Figure 1:**
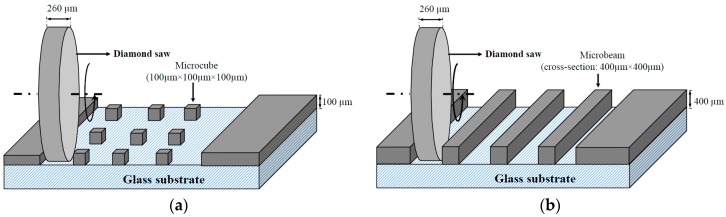
Schematic view of sample generation: (**a**) microcubes and (**b**) microbeams.

**Figure 2:**
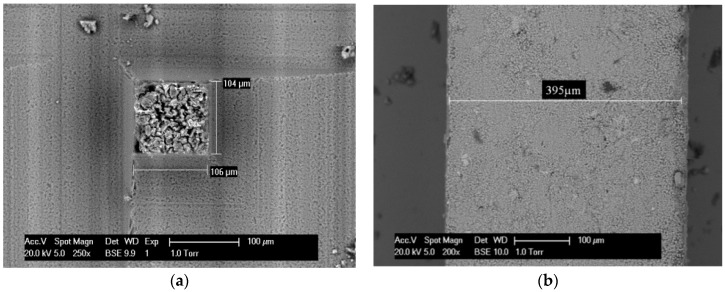
Scanning electron microscope images of single (**a**) microcube and (**b**) microbeam.

To create microcubes and the microbeams, the following procedure is followed: The first step is to make the thickness of the specimens even and equal to 100 µm and 400 µm (for microcube and microbeam creation, respectively) using a Struers Labopol-5 thin sectioning machine; afterwards, the microcubes and microbeams are generated by running a precise diamond saw for semiconductor wafers (MicroAce Series 3 Dicing Saw, with a 260 μm wide blade) in two directions over the thin section as schematically shown in Figure 1. To prevent chipping of the edges of the microcubes and microbeams while cutting, a thin layer of soluble glue was applied on the surface of the thin section, which was later removed by soaking the specimen for a short time in acetone. The final cubes are at the dimension of 100 × 100 × 100 µm^3^ and beams are with a cross-section of 400 × 400 µm^2^ as shown in Figure 2.

### 2.2. Global Micro-Mechanical Performance Using Microcube Indentation

**Figure 3:**
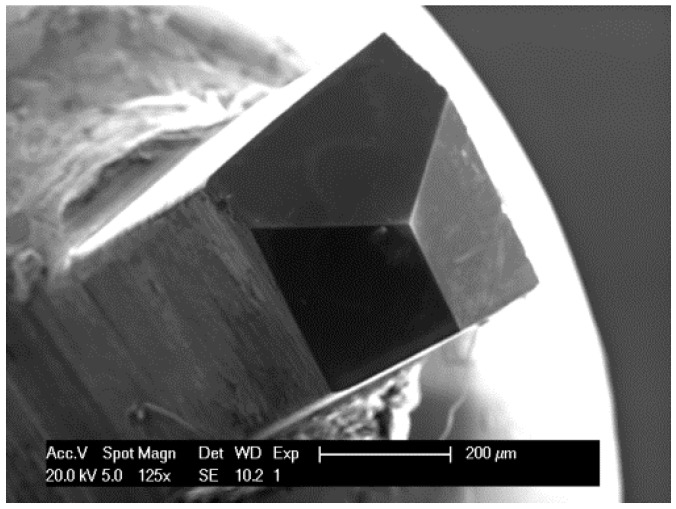
Scanning electron microscope images of diamond Berkovich tip.

**Figure 4:**
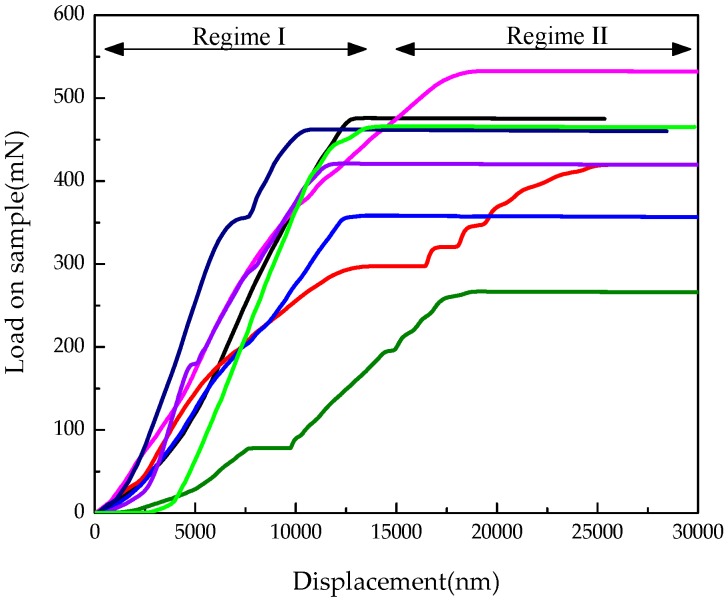
Measured global mechanical response of microcubes: load versus displacement response.

In order to obtain the global mechanical properties, the microcubical cement paste samples were placed in an Agilent G200 nano-indenter and were loaded using a Berkovich tip (Figure 3). A displacement controlled test was performed by using the CSM (Continuous Stiffness Method) [34]. This method relies on applying a small harmonic load with frequency on the nominal load. The CSM settings applied in this study were: 2 nm amplitude, 45 Hz frequency and a displacement rate of 50 nm·s^−1^. More detail about the loading procedure is discussed in [34] in which carbon nano tube bundles are loaded to failure with a flat nano-indenter. The load-displacement response up until failure of the microcube is shown in Figure 4. In total, 8 load-displacements are measured in the experiments for cement paste with a w/c ratio 0.4. Multiple measurements on different cubes show a high degree of repeatability. Two regimes can be distinguished from the paragraph. In regime (I), the load on sample increases monotonically for increasing indenting until reaches the peak load. The maximum load that can be applied before the microcube collapses is between 350 mN and 450 mN at a critical displacement between 10 µm and 15 µm. Once this load is exceeded, the system transitions from a stable regime (I) towards an unstable regime (II) with rapid displacement bursts. The horizontal line in regime (II) indicates structural collapse of the microcube, which results in an overshoot of the nano-indentation tip towards the substrate. Since displacement control of the nano-indenter is not fast enough, it is not possible at present to capture the post-peak behaviour of the specimen. Therefore, the calibration of the numerical model was carried out only in regime (I). It is observed that the test results still show variability which is induced by the inherent heterogeneity of the material.

**Figure 5:**
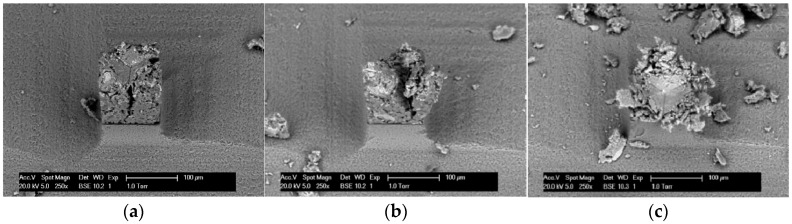
Three stages in the nano-indentation loading process of microcubes observed in ESEM: (**a**) initial stage of loading; (**b**) three main cracks running to the sides of the cubes; (**c**) complete crushing of the sample (adapted from [32]).

The failure mechanism of the specimen under tip loading is observed in previous work [32], where different loading depths were applied. Crack patterns at various depths were obtained using ESEM. As shown in Figure 5, the typical failure mechanism obtained is the crushing of the material under the tip and three main cracks running to the sides of the cubes, starting from the three edges of the Berkovich tip. Complete crushing of the sample was achieved by indenting the tip further into the specimen.

### 2.3. Microstructure Characterization Using of Micro-CT

**Figure 6:**
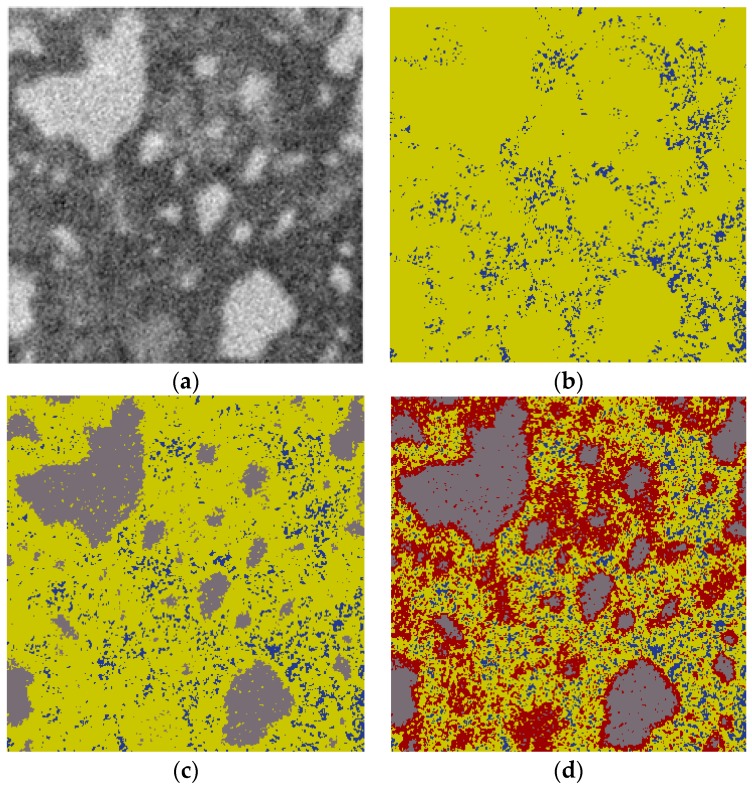
2D Schematic view of image segmentation process: (**a**) original grey-scale map; (**b**) pore (blue) and solid phases (yellow) are isolated from the grey-scale map; (**c**) anhydrous cement (grey) and hydration product (yellow) are isolated form solid phases; (**d**) outer product (yellow) and inner product (red) are segmented from hydration product.

**Figure 7:**
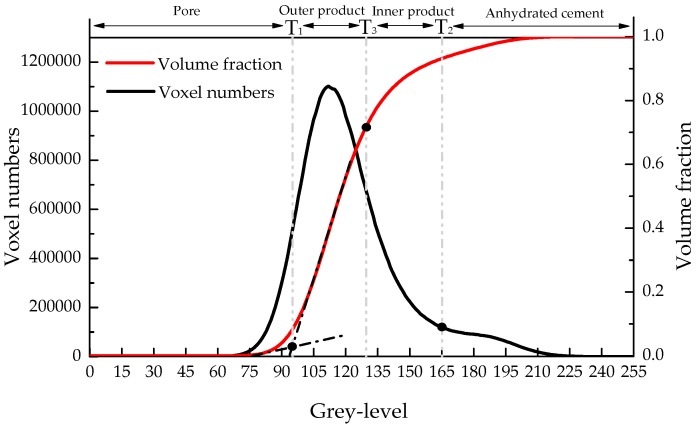
Phases evolution through grey-level histogram of CT images.

In order to obtain the microstructure of the micro-cube, a generated microbeam is scanned using a Micro CT-Scanner (Phoenix Nanotom, Boston, MA, USA). The microbeam was fixed on the holder and then put on the rotatable stage. The X-ray source tube worked at 120 kev/60 mA. 2800 images with an exposure of 6 s were acquired on a digital GE DXR detector (3072 × 2400 pixels). The voxel resolution under these conditions was 0.5 µm^3^. Reconstructed slices were carried out with Phoenix Datos software. For saving the calculation time of mechanical model, the original resolution of reconstructed slices was reduced to 2 µm^3^/voxel. Image segmentation was performed using a so-called global threshold approach [7,9,13,35]. In this method, phases were isolated from the original grey-scale map by choosing the corresponding threshold step by step as shown in Figure 6. Firstly, two threshold grey values are defined on the basis of the grey-level histogram as shown in Figure 7: T_1_, pore/solid phase threshold, is assumed as the grey value at the inflection point in the cumulative fraction curve of the histogram; T_2_, hydration products/anhydrous cement phase threshold, is a critical point at which the tangent slope of the histogram changes suddenly. Three phases can be isolated: pore, anhydrous cement and hydration product. More details about this approach can be found in previous work [13,35]. However, it is well known that at least three types of hydration product with different mechanical properties [14,15,16,17] exist: inner product C-S-H_LD_, outer product C-S-H_HD_ and C-H. In order to simplify procedure, C–H was not considered as a separate phase, and therefore was not explicitly modelled. This simplification is considered not to significantly affect the results of mechanical properties simulation [30]. However, in further work, C-H as a separated phase should be considered.

**Figure 8:**
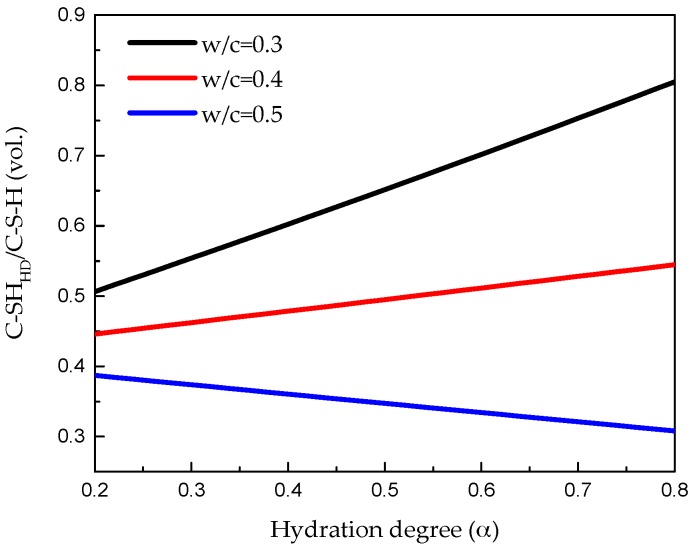
High density Calcium-Silicate-Hydrate (C–S–H_HD_) evolution based on the J-T model [36].

**Figure 9:**
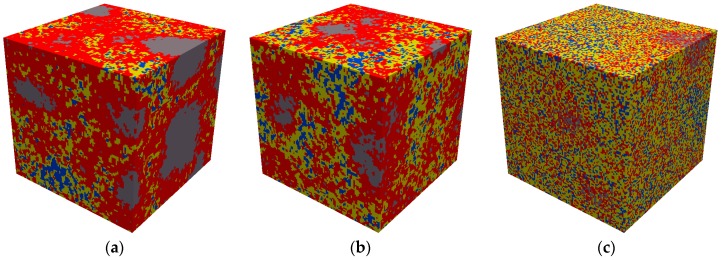
3D segmented microstructure (100 × 100 × 100 µm^3^) of cement paste with w/c ratio (**a**) 0.3; (**b**) 0.4; (**c**) 0.5 (grey-anhydrous cement; red-inner product; yellow-outer product; blue-pore).

A so called J-T model determined analytically by Tennis and Jennings [36] based on specific surface measurements was introduced here to calculate the volume fraction of C-S-H_LD_ and C-S-H_HD_. As shown in Figure 8, the input for this model are w/c ratio and degree of hydration which can be estimated on the basis of volume fraction of anhydrous cement *V*_anhydrous_ and hydration products *V*_hydrated_ according to equation:

(1) $$\alpha =\frac{\frac{V_{\mathrm{hydrated}}}{v}}{\frac{V_{\mathrm{hydrated}}}{v}+V_{\mathrm{anhydrous}}},$$

where *v* is defined as volume reaction product/volume reactant ratio and assumed as 2.2 [37]. Once the volume fraction of those two products is obtained, the threshold value T_3_ can be determined from the cumulative volume fraction cure of grey-histogram as shown in Figure 7. The voxels with a grey value lower than T_3_ are regarded as outer hydration product, while the ones with higher values are inner hydration product. A cubical region of interest with 100 µm in length was extracted from the segmented microstructure for lattice fracture analysis as shown in Figure 9a. Microstructure characterization of cement paste with w/c ratio 0.3 and 0.5 at the same curing age of 28 days were carried out using the same procedure and shown in Figure 9c.

## 3. Modelling

Herein, a microstructure-informed lattice model was calibrated using experimental results of microcube indentation (failure patterns and load displacement diagrams). Then, the global mechanical performance of microcubes with different w/c ratios were predicted by incorporating the microstructure into the lattice model. The modelling approach, calibration and prediction procedure are described in detail below.

### 3.1. Modelling Approach

Again the aim in this study is mainly to determine the micromechanical properties of local phases in lattice model by fitting the experimental results. The lattice model is applied in this study to model the observed fracture behaviour of the tested microcubes. In the model, the material is schematized as a grid of beam elements connected at the ends and all individual elements are defined having linear elastic behaviour. Due to the low ratio of length and height of beam elements in the network, a Timoshenko beam element is used to take shear deformation into account [24]. To achieve the crack growth, unit prescribed displacement is imposed on the lattice system and only one element with the highest stress/strength ratio is removing from the mesh at every loading step. The calculation procedure will repeat several times until a pre-defined failure criterion is reached. This method can express the physical process of fracture behaviour, so that a realistic crack patterns as well as the stress-strain response will be obtained [29]. The “Berkovich tip loading” test is simulated by applying a vertical displacement in four nodes in the centre of the top surface. The lattice element can fail either in tension or compression. The procedure to generate the lattice mesh and assign mechanical properties of elements is as follows.

**Figure 10:**
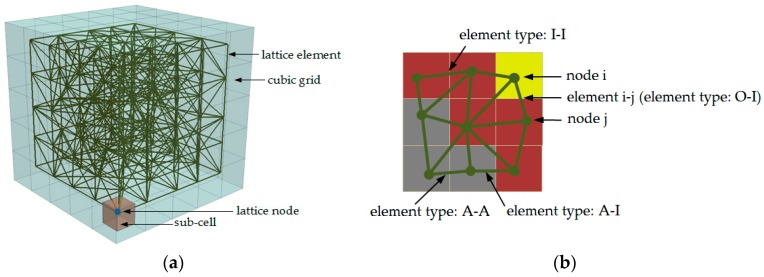
Schematic view of lattice model generation: (**a**) lattice network construction (5 × 5 × 5); (**b**) overlay procedure for 2D lattice mesh (yellow-outer product; red-inner product; grey-anhydrous cement).

The 3D mesh generation is described in Figure 10. First, a cubic domain (100 × 100 × 100 µm^3^) is divided into a cubic grid with a cell size of 2 µm^3^. Then, a sub-cell was defined within each cell in which a node is randomly placed. The ratio between the length of sub-cell and cell is defined as randomness. As shown in preview study [38], the choice of randomness affects the simulated the fracture behaviour of materials, because the simulated crack shape is affected by the orientation of meshes. In order to avoid big variations in length of elements and introduce geometry disorder of material texture, a randomness of 0.5 is adopted. Then, Delaunay triangulation is performed to connect the four nodes that are closest to each other with lattice elements. Afterwards, the cross-section of lattice element is determined by altering this parameter in a homogenous lattice model until the simulated global Young’s modulus matches the assumed local value.

The overlay procedure is applied to realize the heterogeneity of this material. In this procedure, different micromechanical properties are assigned to corresponding phases. For this purpose, the microstructure of cement paste with w/c ratio 0.4 obtained in Section 2.3 is used here. As shown in Figure 10b, each node is assigned a local phase based on the voxel value it belongs to and the lattice element is determined by the locations of its two nodes. No lattice node is generated in the voxels which represent pore phases, as it does not contribute to the global mechanical performance of the specimen. Three solid phases in the microstructure result in six types of lattice elements as listed in Table 1. The shear modulus and Young’s modulus of element i–j connecting phase i and phase j are determined as [30]:

(2) $$\frac{2}{E_{\mathrm{ij}}}=\frac{1}{E_{i}}+\frac{1}{E_{j}},$$

where *E*_i_, *E*_j_ and *E*_ij_, are the Young’s modulus or shear modulus for phase i, phase j and element which connects phase i and phase j, respectively. The compressive strength and tensile strength take the lower value of the connected two phases, which can be expressed in [30]:

*f*_ij_ = min(*f*_i_, *f*_j_), (3)

where *f*_i_, *f*_j_ and *f*_ij_, are the compressive strength or tensile strength for phase i, phase j and element which connects phase i and phase j, respectively. The mechanical properties of these pure phases was preferred to be measured in laboratory test, but in the case of a lack of experimental data, properties of these phases are commonly derived from the nano-indentation measurements [30,31]. However, no data is available defining the relationship between the model parameters (tensile and compressive strength, Young’s modulus) and nano-indentation results (indentation hardness and modulus of elasticity). Herein, in order to work out this relationship, the simulated fracture performance is compared with the experimental results to find out the best simulation, and these values are further used in Section 3.3 to predict the mechanical and fracture properties of hydrated cement pastes with different w/c ratio. Ratios of tensile strength (and compressive strength) among each phase are assumed to be equal to the ratios of measured hardness among these phases in this calibration. For individual phases, values reported in [14] (Table 2) were used in this work. Since scanning electron microscope was adopted to reflect phases at the tested location for the statistics analysis, this method gives more reliable results.

### 3.2. Calibration and Discussion

**Figure 11:**
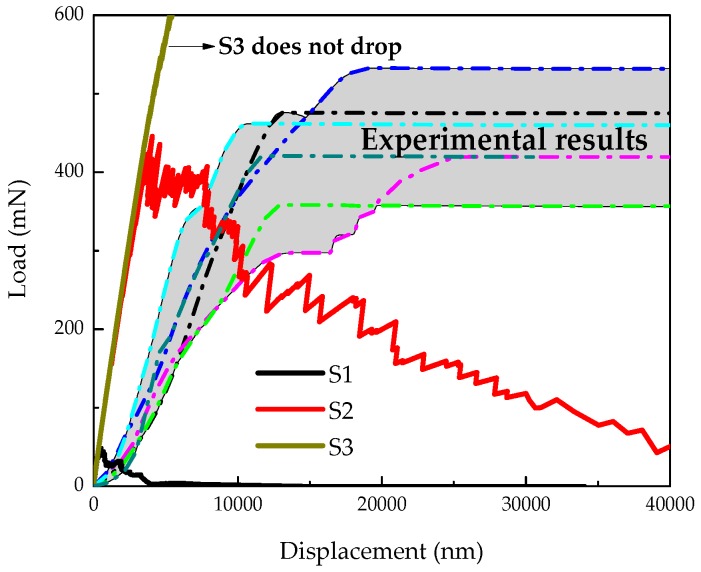
Comparison between simulated load displacement diagrams and experimental results of cement paste with w/c ratio 0.4.

**Figure 12:**
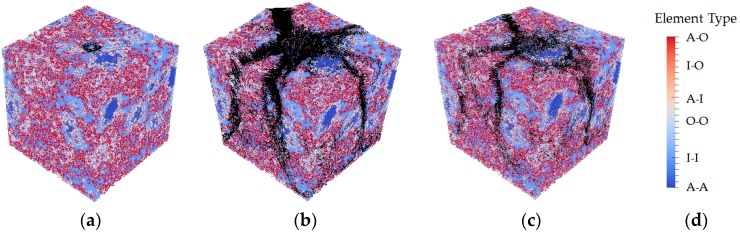
Crack patterns in the final failure state: (**a**) simulation of S1; (**b**) simulation of S2; (**c**) simulation of S3; (**d**) element types (black-damaged element).

During the calibration of mechanical properties of local phases, three sets of parameters as listed in Table 3 were evaluated to study their influence on the simulated fracture performance. The simulated load displacement diagrams are compared with the experimental results as shown in Figure 11. A descending branch is observed in regime (II) for the simulated results, which is missed in the microcube indentation measurement. Their corresponding damaged patterns in the final stage are presented in Figure 12.

The adopted compressive strength/tensile strength ratio of local elements has significant influence on simulated load displacement diagram and damage evolution. Figure 12a shows the damage evolution of S1 in which the compressive strength is taken as 10 times higher than the tensile strength (similar with the assumption in [39]). A few microcracks are initiated around the loading points causing local crushing of the indented area, thus inducing an unrealistically low load response. On the contrary, in the simulation of S3, no softening branch is found in the simulated load-displacement diagram even when the “indenter” reaches the bottom of the microcube specimen. This is because no compressive failure is allowed for local elements in this particular case. In the fracture model, the loading points starts out dragging the upper surface down, and apparent load that can be withstood by the specimen becomes unrealistically high.

Simulation of S2 and “measured” cures show a very high degree of consistency on the peak load and stiffness (slope of the load displacement cure). Due to the fact that some slip occurs at the beginning of the experiments, the measurements are slightly shifted, but the slope is similar to the one in the simulated load displacement curve of S2. In S2, the assumed tensile strength is 12 times lower than the measured hardness, in accordance with [39,40], while the ratio between compressive strength and tensile strength (100) is much higher than in [39]. This may be because of the different resolutions and theories applied in these two models.

### 3.3. Tensile Strength Prediction and Discussion

**Figure 13:**
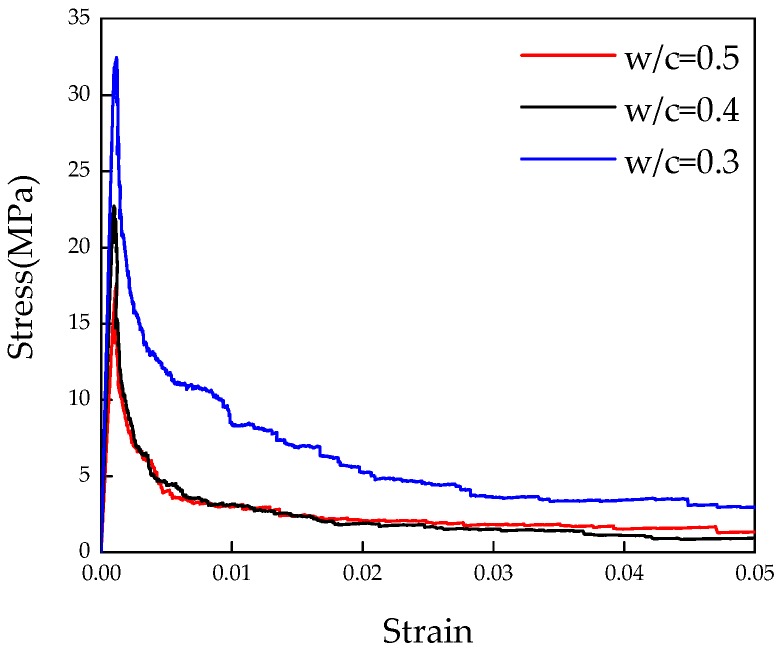
Simulated stress-strain diagrams of cement paste with various water/cement ratios.

**Figure 14:**
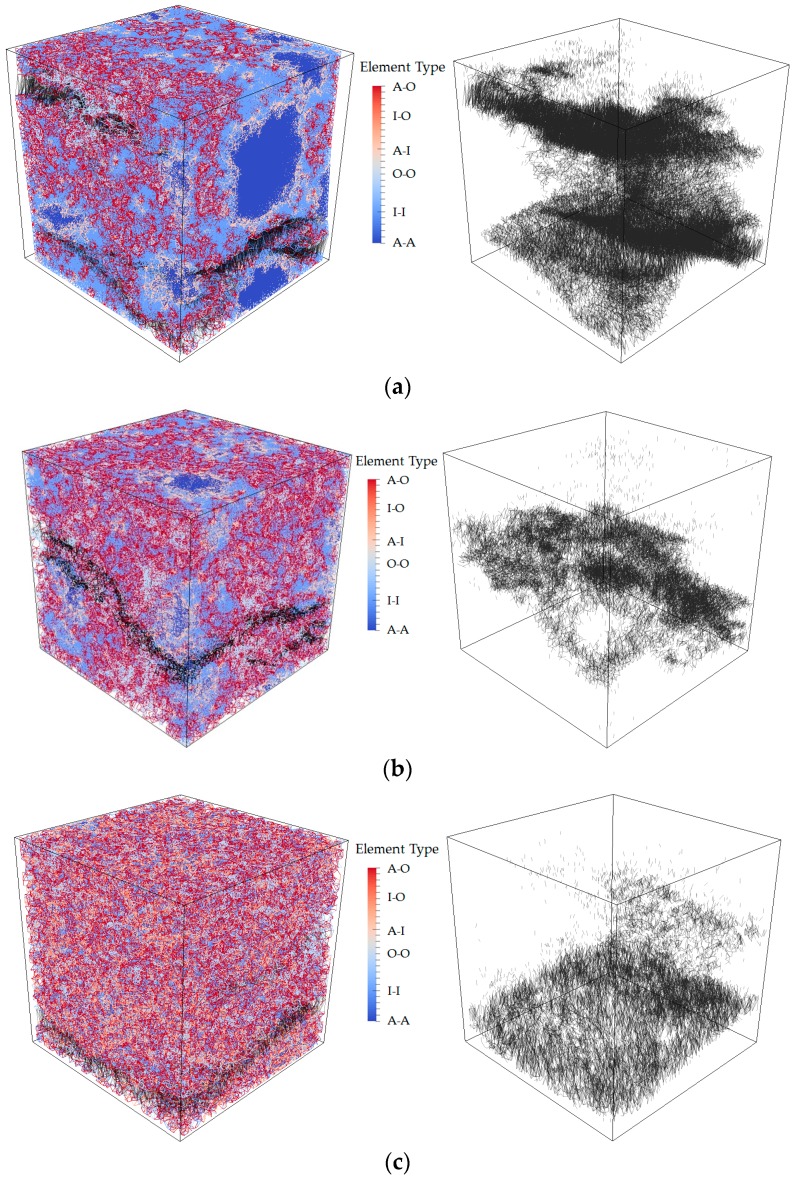
Crack patterns in the final failure state of cement paste with w/c ratio (**a**) 0.3; (**b**) 0.4; (**c**) 0.5 (left: whole specimen; right: only damage).

**Figure 15:**
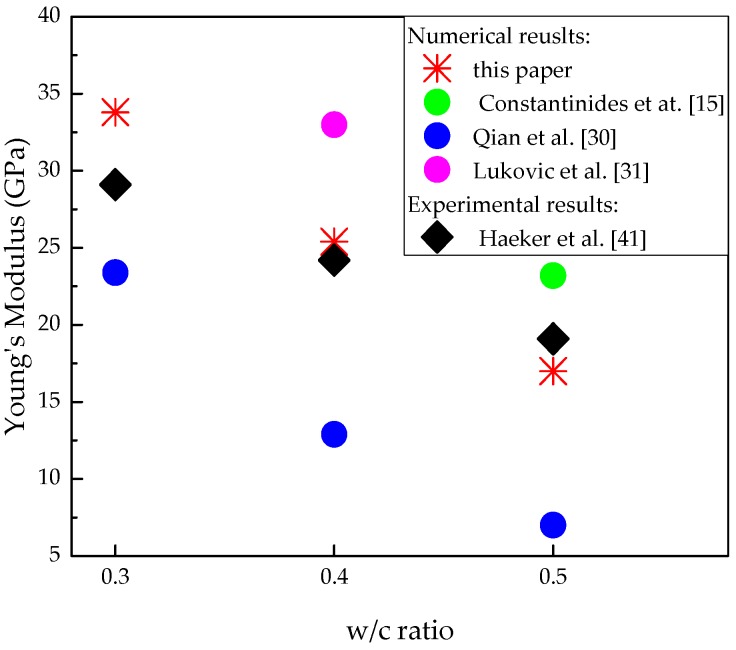
Comparison between predicted Young’s modulus of cement paste and experimental results.

The 3D lattice fracture model is applied on the obtained voxel-based digital microstructures of cement paste to evaluate the mechanical performance under uniaxial tension. The same mesh described in Section 3.1 is applied here. Microstructures with different w/c are applied for the overlay procedure to investigate the influence of w/c ratio on the global performance of cement paste at microscale. The specimen is loaded by applying uniform displacement at one surface, while the opposite surface is clamped. The lattice elements on the surfaces where boundary conditions are applied are not allowed to be broken during the fracture, processes as the external load requires a path into the specimen. The adopted parameters in S2 are assumed as the mechanical properties of local phases. The simulated stress-strain diagrams with corresponding damage pattern are shown in Figure 13 and Figure 14. Mechanical properties parameters are calculated based on the stress-strain curve, and are given in Table 4 together with the previous results. For a w/c ratio 0.4, the calculated results show a good agreement with the results from [30,31]. Since no experimental data of tensile strength is available for comparison at this scale [2], only the simulated Young’s modulus is compared with the experimental results [41] as shown in Figure 15. It can be seen that the simulated Young’s modulus corresponds well with the results from experiments at each w/c ratio. 

A more localized microcracks pattern is observed in the cement paste with higher w/c ratio, which results in a decrease of Young’s modulus and tensile strength. This is because more anhydrous particles are embedded in cement paste with lower w/c ratio and anhydrous particles, as local stiff inclusions, enable more overlaps and branches, which results in a more stable crack development and less brittle fracture. Similar observation is presented in a higher scale of concrete, due to the influence of aggregate [42,43].

## 4. Conclusions

This work presents an approach for the fitting and validation of micromechanical modelling of mechanical properties and fracture mechanism of cement paste. A procedure for micro specimen preparation and testing of global performance using nano-indenter was developed and employed. As a result, fracture pattern under different loading depth and load displacement diagrams were obtained. Two regimes were observed experimentally in the load displacement diagram. Since the displacement control of the nano-indenter equipment is not fast enough to capture the fast decrease in load when the specimen fails, a horizontal line existed in regime (II). Thus, the ascending branch in regime (I) was applied to calibrate the microstructure-informed lattice model. The softening regime was observed in the modelling result.

The 3D lattice model was built up based on a microstructure of cement paste obtained from X-ray computed tomography. The mechanical properties of local phases were calibrated by validating the simulated results with the experimental results. It is shown in this study that the adopted mechanical properties of local phases are critical for the investigation in terms of load displacement response and failure mechanism. Therefore, it is of great importance to fit these parameters by designing experiments on the specimens in the same size as well as under well-controlled boundary conditions.

Furthermore, the calibrated lattice model was applied to predict the fracture performance of the micro cement paste cubes with various w/c ratios under uniaxial tension. Since an experimental method was applied to calibrate the input mechanical properties of local phases, the proposed method in this paper gives more reliable prediction results. The predicted outcome can be used as an input in the multi-scale modelling of the failure mechanism in cement based materials in further study. The method adopted in this study also illustrates a basic investigation for future research to build upon, and an integrity system for the upscaling of the modelling and validation on every scale.

## Figures and Tables

**Table 1:** Classification of lattice element types.

Element Type	Phase 1	Phase 2
A–A	Anhydrous cement	Anhydrous cement
I–I	Inner product	Inner product
O–O	Outer product	Outer product
A–I	Anhydrous cement	Inner product
I–O	Inner product	Outer product
A–O	Anhydrous cement	Outer product

**Table 2:** Measured mechanical properties of individual local phases from [14].

Phases	Modulus of Elasticity (GPa)	Hardness (GPa)
Anhydrous cement	99.2	8.24
Inner product	31.6	1.14
Outer product	25.2	0.75

**Table 3:** Assumed local mechanical properties of individual local phases in cement paste.

Set	Anhydrous Cement			Inner Product			Outer Product		
	*E* (GPa)	*f*_t_ (GPa)	*f*_c_ (GPa)	*E* (GPa)	*f*_t_ (GPa)	*f*_c_ (GPa)	*E* (GPa)	*f*_t_ (GPa)	*f*_c_ (GPa)
S1	99.2	0.683	6.830	31.6	0.092	0.92	25.2	0.0583	0.58
S2	99.2	0.683	68.3	31.6	0.092	9.2	25.2	0.0583	5.8
S3	99.2	0.683	∞	31.6	0.092	∞	25.2	0.058	∞

**Table 4:** Calculated global mechanical properties, compared with previous work.

w/c Ratio	Strength (MPa)	Young’s Modulus (GPa)
0.3	32.9 (42.1 [30])	33.8 (23.4 [30])
0.4	22.3 (20.3 [30]; 19.3 [31])	25.4 (12.9 [30]; 33.0 [31])
0.5	17.3 (10.6 [30])	17.0 (23.2 [15]; 7.0 [30])
